# Determination of Nanoindentation Behavior of HAZ on Glass Material Machined via ECSM Process through Simulation Approach

**DOI:** 10.3390/ma15175870

**Published:** 2022-08-25

**Authors:** Tarlochan Singh, Sahil Sharma, Sarabjeet Singh Sidhu, Evgeny Sergeevich Shlykov, Timur Rizovich Ablyaz

**Affiliations:** 1 Product and Industrial Design Department, Lovely Professional University, Phagwara 144402, India; 2 Mechanical and Industrial Engineering Department, Indian Institute of Technology Roorkee, Roorkee 247667, India; 3 Mechanical Engineering Department, Sardar Beant Singh State University, Gurdaspur 143521, India; 4 Mechanical Engineering Faculty, Perm National Research Polytechnic University, 614000 Perm, Russia

**Keywords:** indentation, hardness, modeling, HAZ, ECSM

## Abstract

The current study develops a numerical model to investigate the nanoindentation behavior of heat-affected zones (HAZ) on glass material produced via the electrochemical spark machining (ECSM) method. Initially, microchannels were created using the ECSM method on soda–lime glass. Following that, a nanoindentation test was conducted to quantify the Young’s modulus and hardness of the glass sample. After that, a numerical model based on finite elements was created to characterize the changes in mechanical characteristics of HAZ. According to the findings, increasing the electrolyte concentration from 10 to 30% increases the intensity of electrochemical discharges, and thereby decreases the hardness of the work material by 16.29 to 30.58% compared to unmachined glass. The results obtained from the simulation are in close agreement with the experimental values. The maximum error obtained between simulation and experimental results is only 4.18%.

## 1. Introduction

**Figure 1:**
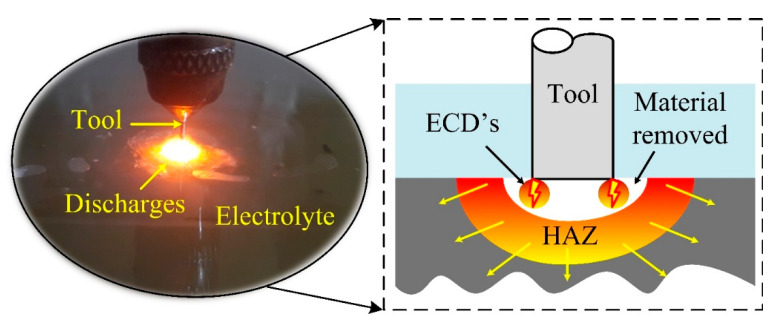
Energy channelization in ECSM process.

Electrochemical spark machining (ECSM) is a hybrid micromachining technique that has been widely employed to create microfeatures in glass for MEMS and microfluidic applications [1]. Owing to the simplicity and small size setup, this process has been extensively used to fabricate intricate microchannels [2,3], micro holes [4,5], and micro slits [6] in both nonconductive and conductive materials [7,8]. The machining setup of ECSM consists of two working electrodes, namely auxiliary electrode (anode) and tool electrodes (cathode), which are commonly submerged in alkaline electrolytes [9,10]. The heat energy generated by the cathode in the form of electrochemical discharges (ECDs) in the ECSM process melts and vaporizes the work surface material [11,12]. Additionally, ECDs increase the temperature of electrolytes, and thus accelerate the electrochemical etching. The combined action of ECDs and electrochemical etching results in a 30-40% higher machining rate than electrochemical machining (ECM) and electro discharge machining (EDM) [13,14]. The high-intensity ECDs originated from the tool electrode changes in the microstructure around the machined microfeatures, and thus altered the mechanical properties, as shown in Figure 1. Sabahi et al. first determined these changes in glass material by performing a nanoindentation test [15]. They found that the area around the microfeatures softens as the magnitude of input energy increases, consequently decreasing the hardness of the material. The area around the feature with reduced hardness was nominated as the heat-affected zone (HAZ). In another investigation, Sabahi et al. obtained a similar result while investigating the characteristics of glass work material after machining with an surfactant-mixed electrolyte in ECDM process through a nanoindentation test [16]. Apart from this, the nanoindentation test can ascertain the elastic modulus and hardness of various materials processed through electric discharge machining, grinding, and polishing processes [17,18]. The reason for using the nanoindentation method over other techniques is its advantages of a small sample space requirement and its independence from the feature geometry [19].

In order to save time and experimental costs, numerical models based on finite elements for several engineering problems have been developed in the last few decades [20]. As per the concern of modeling the nanoindentation test, Karimzadh et al. developed a Finite element modeling (FEM)-based model to predict the Young’s modulus and hardness of aluminum 1100 [21]. In this investigation, two different types of indenters, namely a sharp tip indenter and a round tip indenter, were used, and it was found that the simulation results of the round tip indenter were consistent and in close agreement with the experimental values. Roy et al. modeled the nanoindentation behavior of HAZ and recast the layer of hemispherical features machined via a reverse EDM process, reporting an 11% error between the simulated hardness value and the experimental value [22]. Wagih and Fathy used a 2D axisymmetric FEM model to predict the nanoindentation behavior and distribution of stresses and hardness of Al-5wt%/Al_2_O_3_ nanocomposites [23]. The outcomes exhibited by these models reveal that FEM is an appropriate technique for determining the hardness of material without investing experimental efforts and cost. To date, the numerical model to predict the hardness of glass material processed via the ECSM process has not yet been reported. Thus, in the present manuscript, a 2D axisymmetric FEM-based model is developed to determine the change in glass material hardness processed via the ECSM technique at different energy levels.

## 2. Methodology

**Figure 2:**
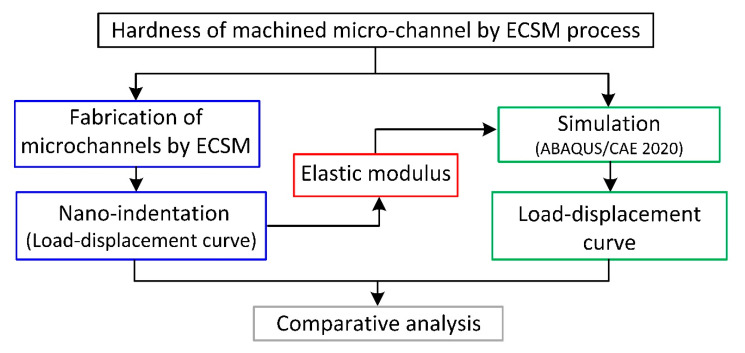
Workflow plan.

In the current investigation, the nanoindentation behavior of HAZ on ECSM-machined microchannels was analyzed. This work is accomplished in two phases, as shown in Figure 2. In the first phase, microchannels were machined via the ECSM process. Subsequently, the values of Young’s modulus and hardness were determined by a nanoindentation test performed on a TI 900 TriboIndenter. In the second phase, a finite element-based model was created to determine the hardness value through a load–displacement curve. In this model, the value of Young’s modulus was used as received from the experimental results, and the rest of the material properties, such as Poisson’s ratio and density, were kept constant. The Young’s modulus values obtained from the nanoindentation test are tabulated in Table 1.

### 2.1. Experimental Procedure

**Figure 3:**
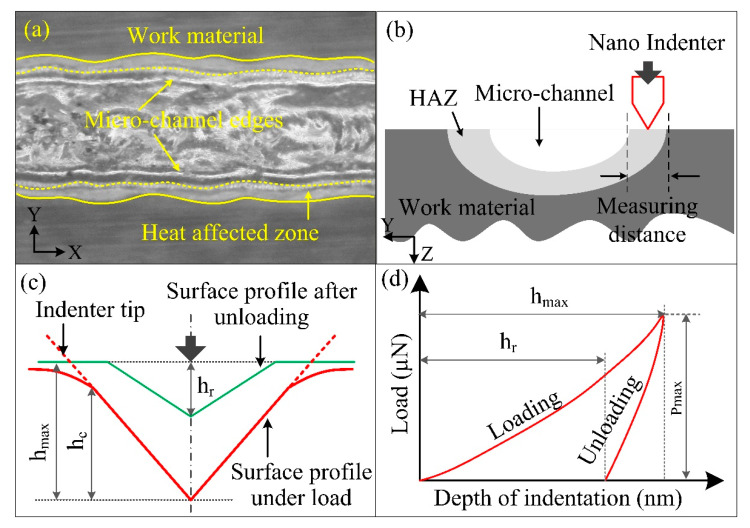
(**a**) Microchannel machined via ECSM process, (**b**) measurement methodology for hardness, (**c**) schematic cross-sectional representation of impression of indenter in work surface, and (**d**) load–displacement curve.

The fabrication of microchannels was carried out on the in-house-constructed ECSM setup. The specifications of the same are reported in Reference [24]. In this investigation, microchannels were fabricated on soda–lime glass by varying the electrolyte concentration of NaOH from 10 to 30%. The remaining parameters were kept constant: electrode diameter = 400µm, applied voltage = 58V, feed rate = 4mm/min, and pulse on-and-off ratio = 3:1. The microscopic image of the microchannel presented in Figure 3a revealed that up to a distance of 100 µm from the side edges of the channel, the microstructure of glass was changed. This area was identified as a heat-affected zone (HAZ). Thereafter, a nanoindentation test was conducted in this area, as shown in Figure 3b.

The test was carried out through the displacement mode by providing a maximum depth of 145 nm. The depth was selected to diminish the effect of surface roughness, and secondly, in the case of soda–lime glass, the impact of the load exhibited almost consistent results after the depth of 145 nm.

A Berkovich indenter was used in this investigation, and it was required to penetrate along the longitudinal direction for ten seconds. Afterward, the indenter was held constant at a maximum depth for the next five seconds. Lastly, the indenter was unloaded to its original position within the next five seconds. The unloading of the indenter left some impressions over the work material. The cross-sectional view of the indentation and the various indentation parameters used in the simulation are represented in Figure 3c. Here, h_max_ and h_c_ represent the depths at the peak loading and at the surface of the indenter’s perimeter, respectively, during loading condition. During unloading, as the indenter is removed completely from the work surface, the elastic displacement of the work surface is recovered, leaving the impression of depth indicated by h. The load versus displacement (LVD) curve obtained after the completion is represented in Figure 3d.

The outcome was considered as the average of ten indents performed at different locations, and the hardness of HAZ and parent material was calculated by the equations [25] given below:

(1) $$H=\frac{P_{\max}}{A}$$

(2) $$A=\left(3\sqrt{3}\tan^{2}\theta \right)h_{c}^{2}$$

where *P*_max_ is the load at maximum indentation, *A* is the projected area of indentation, *θ* is the half-angle of the indenter, and *h_c_* is the contact depth, which is defined as the distance in the longitudinal direction along which the contact is established. The *h_c_* can be determined as follows:

(3) $$h_{\max}=h_{c}+h_{a}$$

(4) $$h_{a}=\frac{\Pi -2}{2}\times h_{e}$$

(5) $$h_{e}=h_{\max}-h_{r}$$

where *h_max_* and *h_r_* are the depth at maximum loading and unloading conditions that can be directly obtained from the LVD curve, and *h_e_* is the elastic displacement during indenter unloading.

**Figure 4:**
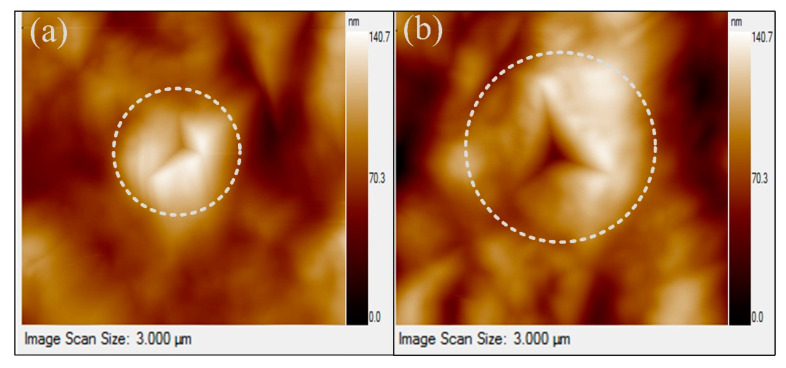
Indenter impression over the (**a**) parent glass material and (**b**) glass machined through ECSM process.

The indenter impressions obtained from the unmachined and machined glass work materials are depicted in Figure 4a,b, respectively.

### 2.2. Simulation Procedure

**Figure 5:**
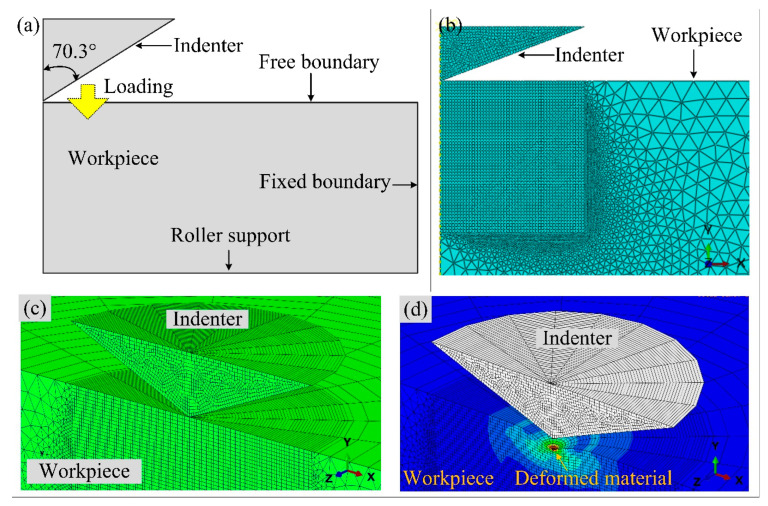
(**a**) Schematic representation of indenter and workpiece with boundary conditions, (**b**) axisymmetric mesh pattern, (**c**) 3D mesh pattern, and (**d**) deformed work material in 3D view.

Abaqus/CAE 2020 was used to mimic the same load–displacement curve through a simulation approach. An axisymmetric 2D model was developed. The properties of material selected for simulation were: density = 2440 kg/m^3^, Poisson’s ratio = 0.23, and initial yield stress = 30 MPa. The values of yield stress and plastic strain used in this study have been tabulated in Table 2. The boundary conditions are illustrated in Figure 5a. The model employed a conical rigid indenter with a 70.3 degree half-angle that exhibits the same projected area-to-depth function as the standard Berkovich indenter [21]. The indenter and work material hard-contact constraint were defined by designating the indenter and work material as master and slave surfaces, respectively. Mechanical static displacement/rotation was used as the loading boundary condition for both the indenter and workpiece. The master surface was allowed to move along the longitudinal direction up to a maximum depth of 145 nm. The indenter motion was specified using the Tabular Amplitude function to simulate the real indenter motion employed in the nanoindentation. Because the master surface comes into contact with the slave’s top surface, a free boundary condition was allocated to the upper surface, while the slave’s bottom and right edge maintained a fixed state. Quadrilateral mesh with very fine global-size elements was selected to mesh the area under the tip of the indenter to accurately predict the stress distribution in the contact area. Meanwhile, to save on computational time, a coarse triangular meshfree technique was adopted for the rest of the workpiece domain, as shown in Figure 5b. For the meshing of a rigid conical intender, axisymmetric linear element meshing was used to minimize the computational cost.

## 3. Results and Discussion

During nanoindentation, the work material is processed under various stages such as loading, holding, and unloading. The work material becomes plastically deformed when the applied equivalent stresses (*σ_eq_*) generated during the loading stage exceed the material yield strength. The following equation may be used to compute the equivalent stress:

(6) $$\sigma_{eq}=\frac{{\left(\sigma_{\alpha }-\sigma_{\beta }\right)}^{2}+{\left(\sigma_{\beta }-\sigma_{\gamma }\right)}^{2}+{\left(\sigma_{\gamma }-\sigma_{\alpha }\right)}^{2}}{2}$$

where, *σ_α_*_,_ *σ_β_*_,_ and *σ_γ_* are the three principal stresses.

**Figure 6:**
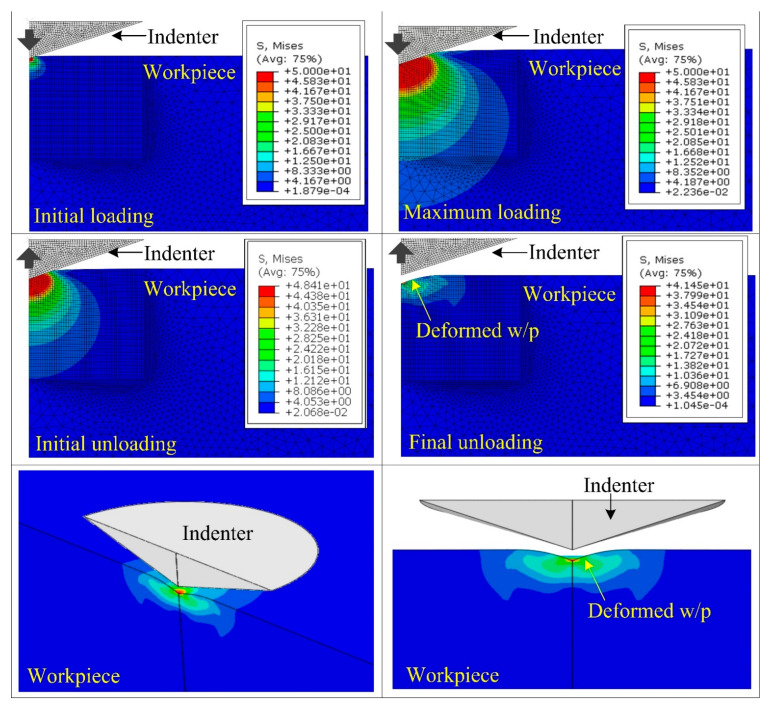
Stress contours to represent nanoindenter behavior for different stages.

The simulation also provides the stress contours in the determination of equivalent stresses, as shown in Figure 6. As the indenter comes in contact with the work material, the value of equivalent stresses increases immediately below the indenter tip. In the loading stage, the value of equivalent stresses is 50 MPa, which is higher than the glass work material yield strength. As a result, plastic deformation occurs instantly when the indenter tip makes contact with the sample surface. With a further increase in indentation depth, the value of equivalent stress increases in horizontal and vertical directions until the indenter penetrates up to 145 nm depth, as shown in Figure 6. During the unloading stage, the equivalent stresses start decreasing as the indenter detaches from the work material. Hence, the material recovers elastic deformation, and due to the presence of residual plastic deformation, the indenter tip produces impressions on the work surface [26].

**Figure 7:**
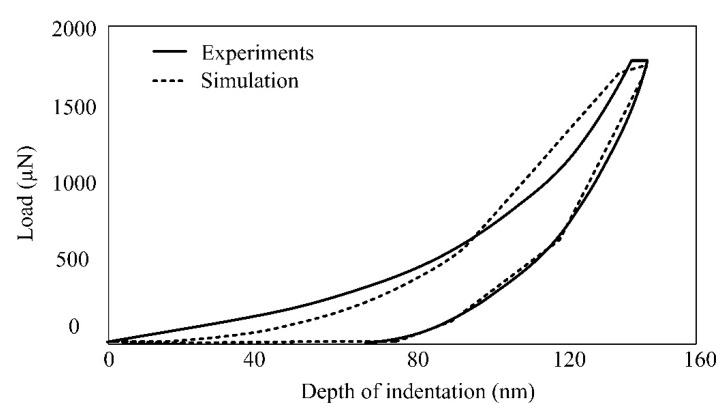
Load–displacement curve obtained from experimentation and simulation.

The LVD curves obtained from experimentation and simulation are represented in Figure 7. The close agreement between experimental and simulation findings demonstrates the FE model’s accuracy in predicting the mechanical characteristics of machined glass material using the ECSM process.

**Figure 8:**
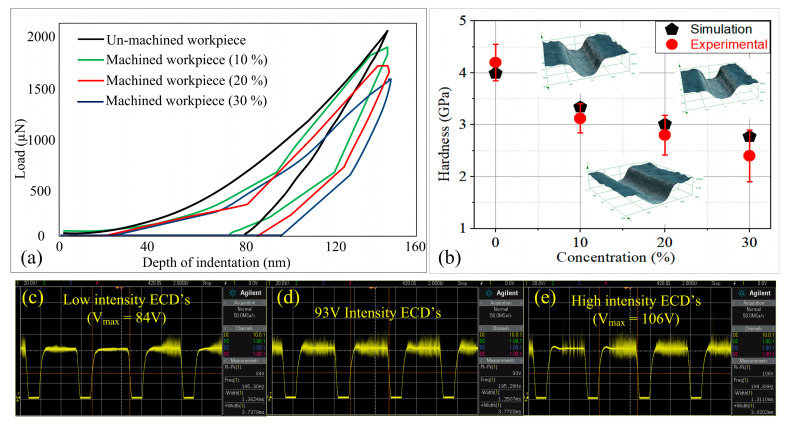
(**a**) Load–displacement curves obtained from simulation results, (**b**) comparison between simulation and experimental results; discharge signals obtained at (**c**) 10%, (**d**) 20%, and (**e**) 30% electrolyte concentration.

By following this procedure, the simulations have been carried out for different samples of glass material machined at different electrolyte concentration levels, and the respective load–displacement curves are shown in Figure 8a.

It was observed that the maximum load required to penetrate the indenter for the unmachined glass material is 2079.65 µN. As the electrolyte concentration increased from 10 to 30%, the maximum indentation force decreased gradually. Therefore, the glass hardness was found to decrease by 16.29, 24.56, and 30.58% compared to unmachined glass material, as shown in Figure 8b. The reduction in hardness with the increasing electrolyte concentration demonstrated that ECSM processing softens the work material. This can be understood from the variations obtained in the intensity of the electrochemical discharges (ECDs) for different electrolyte concentrations (Figure 8c–e). The intensity of ECDs is represented by the term Vmax. The increase in electrolyte concentration reduces the interelectrode resistance, which in turn increases the intensity of the electrochemical reactions [15]. Consequently, a thick gas film forms around the cathode, and therefore generates high-intensity electrochemical discharges (ECDs). The increase in thermal energy owing to spark intensity conducts more heat in the surrounding area of the machined features, and thereby softens the microstructures of the glass work material.

To validate the developed FEM-based model, the simulation and experimental results were compared as depicted in Figure 8b. From this figure, it can be evidenced that the simulation findings were in close agreement with the experimental results. The maximum error obtained was 4.18%, which shows the effectiveness of the presented model in predicting the nanoindentation behavior of glass material.

## 4. Conclusions

This work was accomplished to create a FEM-based axisymmetric model for determining the nanoindentation behavior of the heat-affected zone (HAZ) of soda–lime material processed via the ECSM technique. Initially, microchannels were fabricated in the glass by varying the electrolyte concentration from 10 to 30%. Subsequently, the change in hardness due to the ECSM machining was determined by nanoindentation testing. Thereafter, a FEM-based model was developed to predict the hardness of material in HAZ. In this model, the load–displacement curve and hardness were obtained as response characteristics. The simulation results were validated and compared to the nanoindentation results. The obtained results revealed that the maximum error in hardness was only 4.18%, which signifies the validation of the presented model for predicting the change in properties of ECSM-machined glass. Moreover, the obtained load–displacement curve was found to be consistent with and in close approximation to the curve reported from the nanoindentation. Apart from this, it was obtained that the hardness of material decreased by 16.29, 24.56, and 30.58% when machined at 10, 20, and 30% electrolyte concentration, respectively, compared to unmachined glass. The reason attributed to this softening behavior was the origination of high energy in the vicinity of the tool and the workpiece.

## Figures and Tables

**Table 1:** Young’s modulus obtained from nanoindentation test.

Glass machined at different electrolyte concentration	0%	10%	20%	30%
Young’s modulus (GPa)	76.84985	75.63827	74.38144	72.1095

**Table 2:** Variation in plastic strain with yield stress.

Yield Stress (MPa)	30	42	50	55
Plastic strain	0.00	0.021	0.046	0.051

## Data Availability

Data are contained within the article.
